# Identification of Aberrantly Expressed lncRNAs Involved in Orthodontic Force Using a Subpathway Strategy

**DOI:** 10.1155/2019/9250129

**Published:** 2019-09-02

**Authors:** Xinxing Guo, Jianning Wang, Jing Chen

**Affiliations:** ^1^Department of Orthodontics, Jinan Stomatological Hospital, Jinan 250001, China; ^2^Department of VIP Dental Service, Jinan Stomatological Hospital, Jinan 250001, China; ^3^Department of Nursing, School of Stomatology, Shandong University, Jinan 250012, Shandong Province, China

## Abstract

**Background:**

The aim of the study was to identify key long noncoding RNAs (lncRNA) and related subpathways in the periodontal ligament tissue following orthodontic force.

**Methods:**

We adopt a novelty subpathway strategy to identify lncRNAs competitively regulated functions and the key competitive lncRNAs in periodontal ligament disorders after undergoing orthodontic force. To begin with, patients with orthodontics in our hospital were enrolled in our research. The relationship of lncRNA-mRNA was established through shared predicted miRNA by using the hypergeometric test, Jaccard coefficient standardization, and the Pearson coefficient to determine the valid interaction relationship. After embedding screened lncRNA interactions to pathways, the significant subpathways were recognized by lenient distance and Wallenius approximation methods to calculate the false discovery rate value of each subpathway.

**Results:**

The lncRNA-mRNA intersections including 263 lncRNAs, 1,599 mRNAs, and 3,762 interacting pairs were obtained. The enriched mRNAs were further enriched into various candidate pathways such as the PI3K-Akt signaling pathway. Several subpathways were screened, including the PI3K-Akt signaling pathway, 04510_1 focal adhesion, and p53 signaling pathway, respectively. The network of pathway-lncRNA-mRNA was constructed. Several key lncRNAs including *DNAJC3-AS1*, *WDFY3-AS2*, *LINC00482*, and *DLEU2* were screened.

**Conclusions:**

*DNAJC3-AS1*, *WDFY3-AS2*, *LINC00482*, and *DLEU2* as aberrantly expressed lncRNAs involved in orthodontic force might play crucial roles in periodontal ligament disease pathogenesis.

## 1. Introduction

Orthodontic tooth movement was considered a response to remodeling processes of the alveolar bone, which are initiated by the application of orthodontic force in the periodontium [1]. Bone remodeling is interceded by the release of cytokines and growth factors in the cellular paracrine environment, which are caused by the transmission of signals from extracellular matrix leading to alteration of nuclear protein matrix and gene activation or suppression [2]. Although there is increasing clarification of the primary aberrant cellular processes responsible for orthodontic tooth movement, the underlying mechanisms of many periodontal ligament disorders are still not fully understood. However, little information is available concerning lncRNAs involved in the field of the periodontal transcriptome [3]. Improved understanding of transcriptome arose by orthodontic forces helps us deepen the understanding of periodontal ligament disease pathogenesis, nominate new biomarkers, and motivate new therapeutic strategies.

Long noncoding RNAs (lncRNAs) have longer than 200 nucleotides regulating gene expression in epigenetics and transcription at the level of posttranscriptional repression of their target genes in a sequence-specific manner [4]. In addition, a tremendous amount of lncRNAs have no biological function annotations although there has been accumulating evidence that lncRNAs are closely associated with regulating various biological and pathological processes in the past 10 years [5]. However, researchers in the field of periodontium incorporated regulatory interaction information between periodontal ligament disease and lncRNAs biology into their studies [6, 7]. lncRNAs have also been implicated in periodontal ligament disease, such as previously periodontal ligament disease-associated *VAMP3* [8] and *CCND2* [9]. However, to our knowledge, orthodontic forces-associated lncRNAs and related functions and pathways in human have little been studied.

According to the competitive endogenous RNA (ceRNA) hypothesis, lncRNAs competitively bind to microRNA sites and regulated mRNAs expression levels [10, 11]. Through microarray profiling and bioinformatics analyses, firstly, lncRNA-mRNA competitively regulated interaction was constructed by the hypergeometric test and Jaccard coefficient. Then, we converted the Kyoto Encyclopedia of Genes and Genomes (KEGG) pathways into undirected graphs where genes as nodes and regulated relations as edges. After matching lncRNA-mRNA interaction to KEGG, lncRNA competitively regulated signal pathways (LRSP) were obtained. Interesting lncRNAs and genes were mapped into LRSP and subpathways were pitched in pathways by the lenient distance similarity method [12]. Eventually, the significance of candidate subpathways was assessed according to the Wallenius approximation [13]. We noticed that the deregulated lncRNAs caused by orthodontic forces represented complex regulation networks and participated in the immune system, signal transduction, translation, periodontal ligament tissue contraction, and other pathways.

## 2. Materials and Methods

### 2.1. Samples and Treatment

A total of 16 cases of adult patients that designed for the extraction of four first molars were selected randomly (8 cases in the treatment group, 8 cases in the control group), including 10 females and 6 males, aged 20–26 years old.

All the patients who participated in the experiment have healthy, noncarious human premolar teeth and informed consent from the hospital. The maxillary first premolar in each patient was stressed by 6 ounces of orthodontic traction interact torsion force value, then taking on the force on the maxillary first premolar; four weeks later, we gained the periodontal ligament tissue from the teeth. This study was approved by the Ethics Committee (the School of Stomatology Shandong University).

### 2.2. Microarray Assay

For the control group, the teeth were extracted individually at the same time similar to the treatment group and periodontal ligament tissue was extracted. mRNA were hybridized to Affymetrix Human U133 plus 2.0 gene chip at Shanghai Biotechnology Corporation.

### 2.3. lncRNA-mRNA Competitively Regulated Interaction Construction

MiRNA-mRNA interactions and lncRNA-miRNA intersections were primarily collected from starBase v2.0, TarBase, mortar base, mir2Disease, and miRecords v4.0. Based on the shared miRNAs, lncRNA-miRNA-mRNA interactions were constructed, from which the candidate lncRNA-mRNA competitively regulated interaction will be selected. Briefly, candidate lncRNA-mRNA interactions were determined by the hypergeometric test of shared miRNAs with false discovery rate (FDR) <0.05 and Jaccard coefficient ranked at the top 20%. Hypergeometric functions were computed based on the following formula:

(1) $$\begin{array}{c} P=1-\overset{x}{\underset{t=0}{\sum }}\frac{\left(\begin{array}{c} K\\ t \end{array}\right)\left(\begin{array}{c} N-K\\ M-\;t \end{array}\right)}{\left(\begin{array}{c} N\\ M \end{array}\right)}. \end{array}$$

### 2.4. lncRNA Coexpression Network Construction

Based on the matched lncRNA and mRNA expression profiles, the Pearson correlation coefficient was used to evaluate the lncRNA-mRNA intersections. After Fisher's *Z* transformation, *P* value < 0.01 was considered to be significant lncRNA-mRNA intersections under specific conditions in our study.

### 2.5. Subpathway Analysis

**Figure 1:**
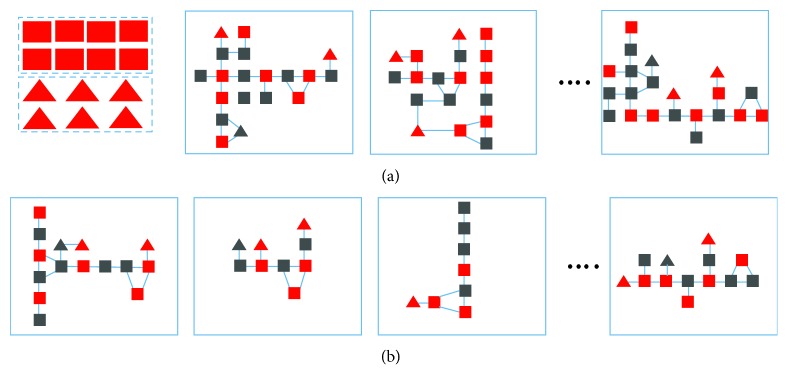
Identification of lncRNA-regulated subpathways. lncRNA: long noncoding RNAs. (a) Map interested mRNA and lncRNA into linked pathway graphs. (b) Identify lncRNA-regulate subpathways.

The lncRNA-regulated subpathway comprised two steps: mapping interested mRNAs and lncRNAs into linked pathways, and identifying lncRNA-regulated subpathways (Figure 1).

#### 2.5.1. Screening of Candidate Different Pathways

mRNAs of lncRNA-mRNA interactions enrichment analysis was carried out based on the KEGG. Different pathways were identified by the Fisher test with the threshold of adjusted *P* < 0.01. In order to reconstruct the lncRNA competitively regulated signal pathways (LRSP), lncRNAs were embedded in the different pathways.

#### 2.5.2. Different Pathways of Embedding lncRNAs

We reconstructed subways graphs by embedding lncRNAs to different pathways. lncRNA and miRNA participating in the competing regulation were deemed important nodes, which could assist us with precisely locating subpathways.

#### 2.5.3. Identification of lncRNA Competitively Regulated Signal Subpathways

Important nodes were linked to LRSP and lenient distance similarity and network topology feature were used to identify the LRSP subways [12]. The detailed processes were described below. Firstly, the shortest path between any two important nodes of LRSP was calculated, if the count of molecules nodes between each pair of signatures was no more than *k* (*k* = 1); then, they will be merged into one node. Next, the number of nodes of the molecule sets in pathways were calculated, whose nodes count was no smaller than *m* (*m* = 8) were considered as candidate LRSP subpathways.

## 3. Results

### 3.1. Screening of Differentially Genes

**Figure 2:**
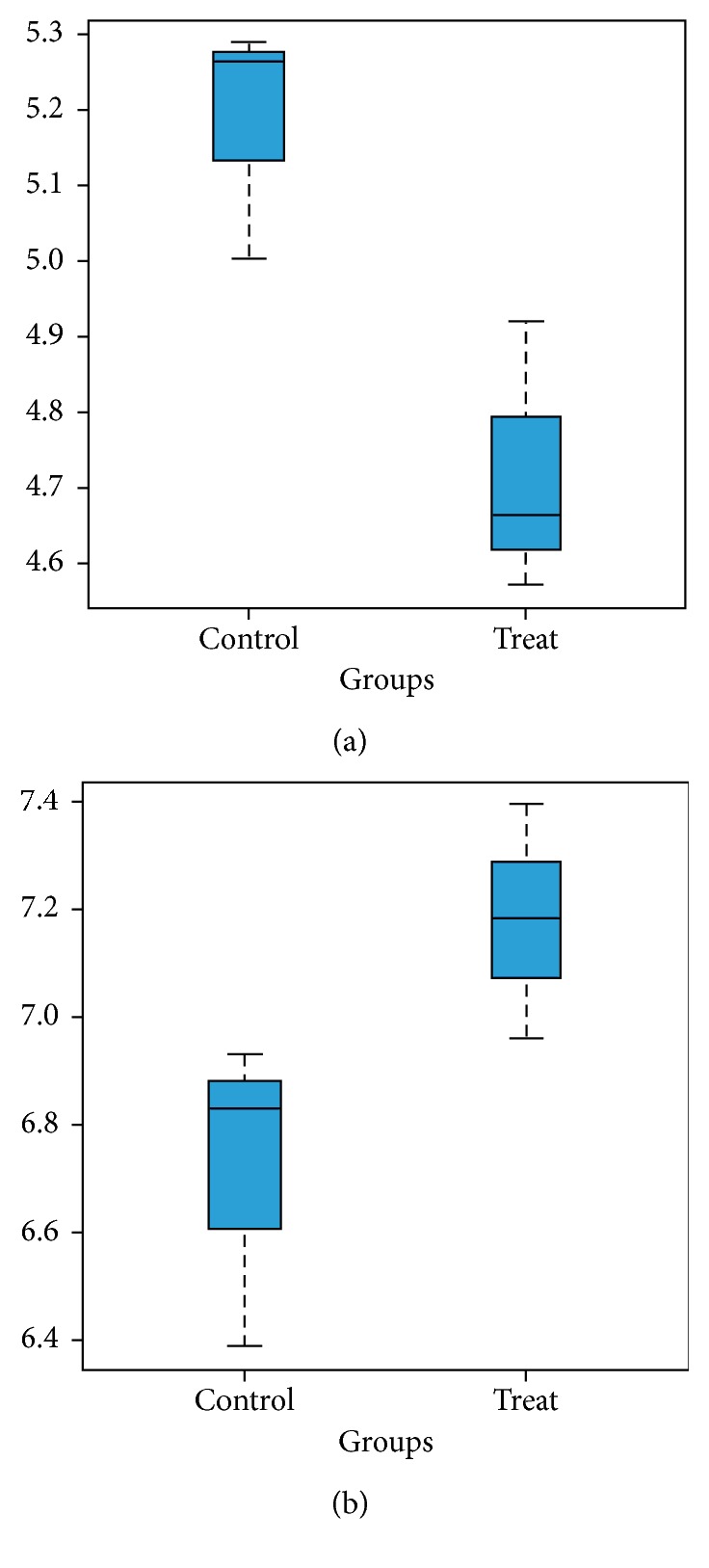
*DNAJC3-AS1* (a) and *DLEU2* (b) expression level between the treatment group and the control group. *Y*-axis represents the gene expression level. *X*-coordinate represents both the control group and the treatment group.

Based on the platform of hgu133plus2, through systematic processing and statistics for the raw microarray data, we obtained the differentially expressed genes and the critical differentially expressed genes (using adjusted *P* value ≤ 0.05). *DNAJC3-AS1* (log fold change = −0.466, *P*.adjust = 0.0142) was downregulated while *DLEU2* (log fold change = 0.464, *P*.adjust = 0.0412) was the most upregulated lncRNA (Figure 2).

### 3.2. lncRNA-mRNA Competitively Regulated Interaction Construction

**Figure 3:**
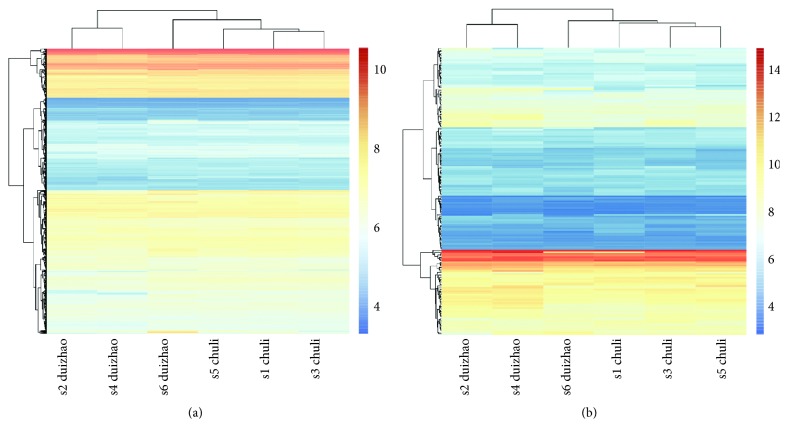
Heat maps showing the distinct lncRNA (a) and mRNA (b) expression profiles between the control group and the treatment group. Red colour represents upregulation; blue colour represents downregulation.

As we know, lncRNAs contained miRNA-response elements and could competitively anchor to miRNAs, indirectly regulating mRNAs [10]. Based on the miRNA-mRNA interactions and lncRNA-miRNA intersections downloaded from multiple RNA databases, the lncRNA-mRNA interaction was obtained through shared miRNAs. A hypergeometric test was executed for each lncRNA-mRNA pair; combined with Jaccard coefficient ranking, the candidate lncRNA-mRNA interactions contained 3762 interaction pairs and 1599 mRNAs, and 236 lncRNAs were obtained. The genes of expression profiling were intersected with mRNA and lncRNA in the lncRNA-mRNA interaction, respectively. Finally, 1502 mRNAs and 169 lncRNAs were screened out for further study (Figure 3). The intersection between RNA expression profiles and candidate lncRNA-mRNA interaction was considered as lncRNA-mRNA competitively regulated interaction.

### 3.3. lncRNA Coexpression Network Construction

In this study, the Pearson correlation coefficient was utilized to calculate the coexpression coefficient for any pair of candidate lncRNA-mRNA interactions. A total of 76 lncGenePairs, 31 lncRNAs, and 71 mRNAs were identified with *P* value < 0.05. The top 5 lncGenePairs were EMX2OS opposite strand/antisense RNA-ACTN3 (*P*=0.0001), THAP7-AS1-C19orf70 (*P*=0.0099), IQCH-AS1-PPFIBP2 (*P*=0.0151), PITPNA-AS1-SOSTDC1 (*P*=0.0296), and EPHA1-AS1-TRAPPC9 (*P*=0.0238) (Table 1).

### 3.4. Sub Subpathway analysis

#### 3.4.1. Screening of Candidate Different Pathways

KEGG is a database resource that integrates genomic, chemical, and systemic functional information. In the present study, we firstly mapped mRNAs of the candidate lncRNA-mRNA interactions to KEGG, with the Fisher test *P* value < 0.01, a total of 8 different pathways were identified (Table 2). The enriched mRNA were enriched into various candidate pathways including pathways in cancer (FDR = 1.14 × 10^−4^), PI3K-Akt signaling pathway (FDR = 4.05 × 10^−4^), and focal adhesion (FDR = 1.06 × 10^−3^).

#### 3.4.2. Different Pathways of Embedding lncRNAs

**Figure 4:**
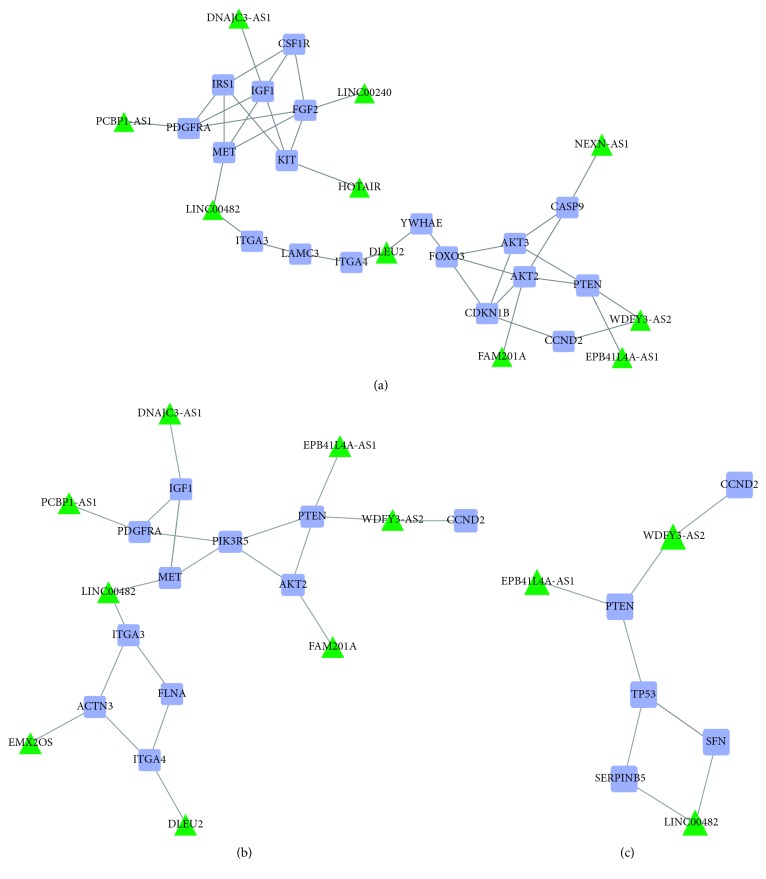
(a) 10 lncRNAs, (b) 8 lncRNAs, (c) 3 lncRNAs participated in potential lncRNA-mRNA interactions. The screened lncRNA and related mRNA were regarded as triangular and quadrilateral nodes. Edges represent relationship between lncRNA and mRNA.

The screened lncRNAs were embedded into candidate pathways according to the results of lncGenePairs. Then, 76 lncRNAs of the candidate lncRNA-mRNA interactions were embedded to the 8 different pathways, and 66 pairs of matched lncRNAs and genes were obtained (Table 3). LRSP included the lncRNA node and edge of lncRNA-mRNA competitive regulation.  Figure 4 shows the predicted form of lncRNA-mRNA interactions.

#### 3.4.3. Recognition of LRSP Subpathways

We reconstructed condition-specific LRSP by embedding lncRNAs to different pathways based on matched lncRNA-mRNA expression profiles and shared miRNA. LRSP subpathways including interesting lncRNA were located to pathways according to the “lenient distance” similarity method. After calculating the significance by Wallenius approximation, LRSP subpathways were ranked (Table 4).

**Figure 5:**
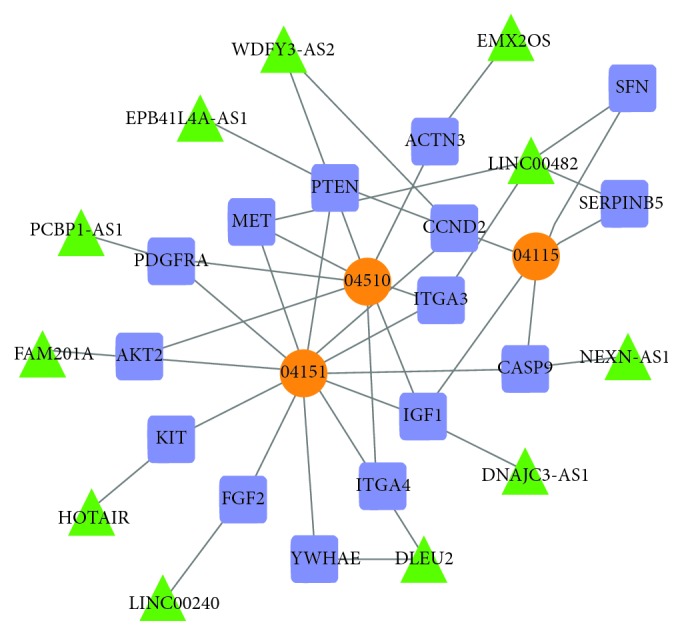
Interaction networks of key lncRNA-mRNA. Square patterns were mRNAs, triangle pattern represents lncRNA, and the circular pattern represents pathway identity. While green triangles were key lncRNAs, an edge represents the regulated relationship between lncRNA and mRNA.

After identification, several subpathways were screened, including the PI3K-Akt signaling pathway, focal adhesion, and pathways in cancer (Table 4). In the top 3 pathways, various lncRNAs and related genes were involved (Figure 5). For example, *LINC00482* in focal adhesion pathway was closely associated with *MET* and *ITGA3*. Besides, insulin-like growth factor-1 (IGF1) involved in the PI3K-Akt signaling pathway, focal adhesion, and p53 signaling pathway. Furthermore, *IGF1* in the PI3K-Akt signaling pathway was associated with *DNAJC3-AS1* and *FAM201A*.

## 4. Discussion

Increasing evidence has shown that lncRNAs were important factors for regulating gene expression [14]. However, the molecular mechanism in lncRNAs driven by orthodontic force including lncRNA functions and regulatory genes remain unknown. In this study, lncRNA-regulated subpathways were identified, including the PI3K-Akt signaling pathway, as well as focal adhesion and prostate cancer signaling pathways. More importantly, several key lncRNAs were identified, including *DNAJC3-AS1*, *WDFY3-AS2*, *LINC00482*, and *DLEU2*. In addition, the subpathways regulated by the lncRNAs and functions of these lncRNAs were predicted, which allowed us to identify lncRNAs of interest that may underline orthodontic forces pathogenesis for further studies.

Insulin-like growth factor-1 (IGF1) in the PI3K-Akt signaling pathway, p53 signaling pathway, and focal adhesion was regulated by DNAJC3 divergent transcript (DNAJC3-AS1). Previous research found that IGF1 was one of the most potent natural activators of the Akt signaling pathway, by stimulating cell growth and proliferation and potently inhibiting programmed cell death [15]. Sant'Ana et al. [16] verified that IGF1 stimulated a mitogenic response and favored the adhesion of PDL cells in vitro, suggesting its possible role in periodontal regeneration. Moreover, IGF1 as growth factor was modulated the response of periodontal ligament cells to inflammation by the orthodontic load. Meanwhile, the cyclic tensile strain of high magnitude significantly inhibited the IGF1 synthesis [17]. Xiang et al. found that *DNAJC3-AS1* has performed dysregulation in human periodontal ligament [18]; this is consistent with our results. Consequently, *DNAJC3-AS1* was important for recovery of periodontal tissue by regulating *IGF1*.

WDFY3 antisense RNA 2 (WDFY3-AS2) was found to regulate two mRNAs via focal adhesion, including phosphatase and tensin homolog (PTEN) and cyclin D2 (CCND2). Both *PTEN* and *CCND2* are protein-coding genes. *PTEN* encoded a phosphatidylinositol-3,4,5-trisphosphate 3-phosphatase [19], the gene *CCND2* belongs to the highly conserved cyclin family, and they were mainly present in cytosol and nucleus. In a previous study, *CCND2* was confirmed to be related to the regeneration of periodontal tissue [20]. As a member of the cyclin family, *CCND2* is a key component for facilitating the G1-to-S-phase transition and subsequently increased cell proliferation. Besides, additional research demonstrated that PTEN might take part in the imbalance between cell proliferation and death [9, 21]. In the present study, *WDFY3-AS2* was enriched into the focal adhesion signaling pathways. Consistent with the study of Molina et al. [22], mechanical stress could bring topographic changes of focal adhesion components and p125^FAK^ activation in stretched human periodontal ligament fibroblasts. Thus, *WDFY3-AS2* served as a potential key lncRNA for the recovery of periodontal ligament cells after the application of orthodontic force by regulating *PTEN* and *CCND2*.

Long intergenic nonprotein coding RNA 482 (LINC00482) was screened as lncRNA in the present study and resulted in the regulation of various genes, such as *ITGA3*, *SNF*, *MET*, and *SERPINB5*, respectively. Integrin, alpha 3 (ITGA3) belonged to the family of integrins and were mainly present in cytosol and cytomembrane. ITGA3 encodes the integrin alpha 3 chains. Alpha chain 3 undergoes posttranslational cleavage in the extracellular domain to yield disulfide-linked light and heavy chains that join with beta 1 to form an integrin that interacts with many extracellular matrix proteins [23]. Saminathan et al. [24] found that mechanical stress reduced the metabolic activity of periodontal ligament cells through effecting the expression of adhesion-related gene *ITGA3*. As we all know that SNF presents in mammalian as SWI/SNF complexes utilizing either Brahma (Brm) or Brahma-related gene 1 (Brg1) catalytic subunits to remodel nucleosomes in an ATP-dependent manner [25]. MET proto-oncogene, receptor tyrosine kinase (MET) associated with multiple diseases (such as lung cancer, hepatocellular carcinoma) in the focal adhesion pathway [26] is a protein-coding gene. Gene ontology notes that *MET* regulates physiological processes including transferase activity, transferring phosphorus-containing groups and protein tyrosine kinase activity. In adults, *MET* participates in wound healing as well as organ regeneration and tissue remodeling in order to promote differentiation and proliferation of hematopoietic cells [27]. Mammary serine protease inhibitor encoded by Serpin family B member 5 (SERPINB5) belongs to the serine protease inhibitor (serpin) superfamily [28]. Serpins regulate a number of cellular processes including phagocytosis, coagulation, and fibrinolysis [29]. Consequently, *LINC00482* may be involved in the mechanism following periodontal ligament cell by regulation of the immune response and inflammation process.

Moreover, it is not difficult to see that deleted in lymphocytic leukemia 2 (DLEU2) directly regulated ITGA4 and YWHAE in the PI3K-akt signaling pathway (see Fig. 5). Yu et al. [30] found that integrin alpha 4 (ITGA4) may play key roles in mesenchymal stem cells derived from periodontal ligament. The product of gene *ITGA4* belongs to the integrin alpha chain family of proteins, like *ITGA3*. Interestingly, this gene encodes an alpha 4 chain associated with either beta 1 chain [31] or beta 7 chains. Tyrosine 3-monooxygenase/tryptophan 5-monooxygenase activation protein epsilon (YWHAE) encoded 14-3-3 protein epsilon belonging to the 14-3-3 family of proteins which mediate signal transduction by binding to phosphoserine-containing proteins [32]. Notably, Abbaszadeh et al. [33] demonstrated that yttrium aluminum garnet laser irradiation on human gingival fibroblasts lead to significantly changed expression proteins produced by *YWHAE*, *UBA52*, *SNF*, and so on. Based on this information, we inferred that *DLEU2* was an important lncRNA for the recovery of periodontal ligament cells after the application of orthodontic force by regulating by regulating *ITGA4* and *YWHAE*.

In conclusion, the strategy of subpathways is feasible to predict marker pathways for periodontal ligament disorders. By integrating the expression profiles of lncRNA and mRNA, several screened lncRNAs including *DNAJC3-AS1*, *WDFY3-AS2*, *LINC00482*, and *DLEU2* in the subpathways of PI3K-Akt signaling pathway, focal adhesion, and prostate cancer might play crucial roles in orthodontic forces pathogenesis.

## Figures and Tables

**Table 1:** The top 10 lncGenePairs.

lncRNA	Gene	corValue	*P* value
EMX2OS	*ACTN3*	9.89 × 10^−2^	0.000159
THAP7-AS1	*C19orf70*	9.17 × 10^−2^	0.009968
IQCH-AS1	*PPFIBP2*	8.97 × 10^−2^	0.01515
PITPNA-AS1	*SOSTDC1*	8.55 × 10^−2^	0.029634
EPHA1-AS1	*TRAPPC9*	8.71 × 10^−2^	0.023883
RAMP2-AS1	*TYW5*	9.72 × 10^−2^	0.001201
H1FX-AS1	*WDR13*	8.24 × 10^−2^	0.043405
PCBP1-AS1	*PDGFRA*	9.58 × 10^−2^	0.002668
PCBP1-AS1	*SIRT1*	9.03 × 10^−2^	0.013484
WDFY3-AS2	*PTEN*	8.86 × 10^−2^	0.018571

**Table 2:** 8 pathways enriched by candidate mRNAs.

	Pathway_names	*P* value	FDR
05200	Pathways in cancer	7.74*E* − 07	0.000114
04151	PI3K-Akt signaling pathway	5.47*E* − 06	0.000405
04510	Focal adhesion	2.39*E* − 05	0.001065
05218	Melanoma	3.76*E* − 05	0.001114
04115	p53 signaling pathway	0.000176	0.004338
05412	Arrhythmogenic right ventricular cardiomyopathy (ARVC)	0.000227	0.00479
05215	Prostate cancer	0.000288	0.005323
05414	Dilated cardiomyopathy (DCM)	0.000512	0.006893

FDR, false discovery rate.

**Table 3:** 10 pairs of matched lncRNA and genes embedded into the 8 different pathways.

Path_name	Matched_lnc	Matched_gene	Path_name	Matched_lnc	Matched_gene
4115	LINC00482	*SFN*	5412	EMX2OS	*ACTN3*
4115	WDFY3-AS2	*PTEN*	5414	LINC00482	*ITGA3*
4151	FAM201A	*AKT2*	5215	DNAJC3-AS1	*IGF1*
4151	WDFY3-AS2	*PTEN*	5218	WDFY3-AS2	*PTEN*
4510	EPB41L4A-AS1	*PTEN*	5200	LINC00482	*ITGA3*

**Table 4:** LRSP subpathways.

Pathway_Id	Pathway_Name	Molecule ratio (m2/*x*)	Molecule list
04151_1	PI3K-Akt signaling pathway	12/69	LAMC3; ITGA3; CSF1R; YWHAE;
			AKT2; IRS1; CCND2; CDKN1B; ITGA4;
			IGF1; CASP9; PDGFRA; KIT; AKT3;
			PTEN; FOXO3; FGF2; MET

04510_1	Focal adhesion	8/69	ITGA3; ACTN3; AKT2; CCND2;
			PIK3R5; ITGA4; IGF1; PDGFRA;
			PTEN; FLNA; MET

05200_1	Pathways in cancer	7/69	ITGA3; GRB2; AKT2; PIK3R5;
			CASP9; PDGFRA; KIT; AKT3;
			PTEN; MET

05215_1	Prostate cancer	5/69	AKT2; PIK3R5; IGF1; CASP9;
			PDGFRA; AKT3; PTEN

04115_1	P53 signaling pathway	4/69	CCND2; SERPINB5; SFN;
			TP53; PTEN

05218_2	Melanoma	4/69	IGF1; PDGFRA; EGF;
			EGFR; FGF2; MET

Id, identity.

## Data Availability

The data used to support the findings of this study are available from the corresponding author upon request.
